# Letter to the Editor from R. McCallum: “Weight Loss From Combination Anti-Obesity Medication Regimens Can Approach That Achieved From Bariatric Surgery”

**DOI:** 10.1210/jcemcr/luad112

**Published:** 2023-09-20

**Authors:** Roland W McCallum

**Affiliations:** Medical School, University of Tasmania, Hobart 7000, Australia; Department of Diabetes and Endocrine Services, Royal Hobart Hospital, Hobart, Tasmania 7000, Australia

Dear Editor,

The success of combination anti-obesity medications (AOMs) makes sense since multiple hormonal pathways are involved in appetite regulation and attacking more than one is likely to give additional benefits [1]. However, when using more than a single agent, attention should be paid to side effects and possible interactions which may be harmful. The following important risks were not mentioned in the impressive case reported by Patel et al [2]:

1. Phentermine and buproprion. Buproprion has the ability to increase cerebral serotonin, which may enter the blood stream. Phentermine inhibits the usually avid uptake of serotonin by red blood cells, thus increasing the risk of higher levels of circulating serotonin known to be associated with heart valve fibrosis [3].
2. Bupropion. This agent has been associated with an increased risk of suicidal ideation in young adults with major depressive or other psychiatric disorders [4]. Therefore, when adding to any regimen, this should be considered.
3. Phentermine and topiramate. An Australian study, which examined the combination of phentermine 15 mg and topiramate 25 mg for weight maintenance, found that 40% of patients could not tolerate it, mainly because of side effects from the topiramate [5]. This is a significantly lower dose than the 100 mg topiramate used in the patient reported.

In summary, while massive weight loss may occur with combinations of multiple AOMs, care should be given to the expert management of pharmacotherapy so that unintended consequences do not occur.

## Disclosures

I have received honoraria from NovoNordisk for lectures and advisory boards.
